# Clinical educator self-efficacy, self-evaluation and its relationship with student evaluations of clinical teaching

**DOI:** 10.1186/s12909-020-02278-z

**Published:** 2020-10-07

**Authors:** Brett Vaughan

**Affiliations:** College of Health & Biomedicine, Victoria University, Melbourne, Australia

**Keywords:** Clinical education, Medical education, Osteopathic medicine, Evaluation, Measurement, Educational environment

## Abstract

**Background:**

In a whole-of-system approach to evaluation of teaching across any degree, multiple sources of information can help develop an educators’ understanding of their teaching quality. In the health professions, student evaluations of clinical teaching are commonplace. However, self-evaluation of teaching is less common, and exploration of clinical educators’ self-efficacy even less so. The aim of the study was to evaluate how a clinical educator’s self-evaluation of teaching intersects with their self-efficacy, to ascertain if that matches student evaluation of their teaching. This information may assist in facilitating targeted professional development to improve teaching quality.

**Methods:**

Clinical educators in the osteopathy program at Victoria University (VU) were invited to complete: a) self-evaluation version of the Osteopathy Clinical Teaching Questionnaire (OCTQ); and b) the Self-Efficacy in Clinical Teaching (SECT) questionnaire. Students in the VU program completed the OCTQ for each of the clinical educators they worked with during semester 2, 2017.

**Results:**

Completed OCTQ and SECT were received from 37 clinical educators. These were matched with 308 student evaluations (mean of 6 student ratings per educator). Three possible educator cohorts were identified: a) high clinical eductor self-OCTQ with low student evaluation; b) low clinical educator self-evaluation and high student evaluations; and, c) no difference between self- and student evaulations. Clinical educators in the first cohort demonstrated significantly higher SECT subscale scores (effect size > 0.42) than their colleagues. Age, gender, teaching qualification, and years practicing or years as a clinical educator were not associated with clinical educator OCTQ scores or the SECT subscales.

**Conclusions:**

Targeted professional development directed towards fostering self-efficacy may provide an avenue for engaging those clinical educators whose self-efficacy is low and/or those who did not receive high student evaluations. Given there is no gold standard measure of clinical teaching quality, educators should engage with multiple sources of feedback to benchmark their current performance level, and identify opportunities to improve. Student and self-evaluations using the OCTQ and evaluation of self-efficacy using the SECT, are useful tools for inclusion in a whole-of-system approach to evaluation of the clinical learning environment.

## Background

A more comprehensive picture of clinical educator teaching quality and performance can be developed through the collection and triangulation of data from multiple sources, including students, peers, program administrators and self-evaluation [1–4]. In clinical education, students will typically evaluate their clinical educators at the end of a clinical placement or rotation offering one perspective of teaching quality. Widely used in clinical education, these evaluations serve to provide feedback to the educators, as well as faculty and program administrators [3, 5] in order to maintain and improve teaching quality [6]. Faculty and program administrators are typically interested in this information for the purposes of professional development activities, remediation, teaching awards, promotion, and potentially ongoing employment decisions [4]. Student evaluations of teaching are used extensively in higher education however authors have highlighted significant challenges with their interpretation (i.e. poor construct definition, gender bias, low reponse rates) and use of the results [7–11], particularly when the student perspective is used in isolation. This collective literature suggests data from student evaluations be limited to formative decision-making that is informed by data collected longitudinally and triangulated with other measures of teaching quality [8, 12, 13].

When data about teaching quality are drawn from multiple sources, it is anticipated that the clinical educator will use this data to assist them to improve their teaching. Gathering this data may also stimulate the clinical educator to reflect on their performance, and institute changes to their education practice to improve teaching quality. The ‘self-regulated professional’ [14] engages in this reflective practice cycle as part of daily clinical practice. However, if or how they use self-evaluation in their practice as a clinical educator is less clear with few examples in the literature [1, 6]. Whilst self-evaluation has been shown to have limitations when used in isolation [14–16], if combined with data from external sources [2, 17–20] it can be regarded as *informed self-assessment* [21] and this combined data can be valuable to improve performance. Self-assessment judgements appear to be multifactorial [21], with contextual factors and “underlying tensions” (p. 1212) influencing the use of data from one source over another.

Our understanding of self-assessment is better informed by exploring the external and internal information individuals draw on to inform this judgement [20–22], whilst also acknowledging that this information can be of varying quality [20]. The current study draws on the definition of self-assessment by Eva and Regehr [16] who describe this construct as:

“ … a pedagogical process by which one takes personal responsibility for looking outward, seeking feedback and explicit information from external sources, then using these externally generated sources of assessment data to direct performance improvements” (p.15).

Several studies have investigated the relationship of clinical education self-evaluation data to that generated by learners [1, 3, 6]. These studies suggest there is limited concordance between self- and student evaluations, inferring potential use of differing standards when making quality judgements [23]. This difference in student and self-evaluation appears to stimulate reflection on performance [1], typically for those who under- rather than over-estimate their own performance [6]. Notwithstanding the aforementioned research, feedback from students appears to stimulate self-evaluation [2, 20].

A potential influence or mediator of self-evaluation of performance, amongst other processes, is self-efficacy [14]. Self-efficacy as a construct stems from the work of Bandura [24] and is defined as the self-perceived ability to perform a task, self-monitoring, and to an extent, motivation to persevere when faced with challenges or difficulties with said task. Self-efficacy, however, is task and context-specific [25], and develops through experience with task outcomes, observation of successful or positive performances, feedback and reflection on task performance [26]. In the clinical teaching context, self-efficacy could be considered to be the beliefs of the educator in their ability to facilitate student learning through engaging with alternative educational approaches, tolerance to mistakes and student-centred learning [27]. Various meta-analyses from the wider educational literature have demonstrated small positive, and significant, relationships between self-efficacy and teaching effectiveness [28], self-assessment and self-efficacy [29], and self-efficacy with a commitment to teaching [30] in teachers. However, we know little about the self-efficacy of clinical educators in the health professions context, and how this construct correlates with teaching quality.

Although the construct of *quality of clinical teaching* has not been agreed on in the literature [31] – likely due to its context-specific nature [32] - it broadly incorporates the interpersonal attributes, and teaching approaches utilised, by clinical educators [33], and is a term widely used in the literature [31, 34–38]. Drawing on Beckman, Ghosh, Cook, Erwin and Mandrekar [33], *quality of clinical teaching* in the current study was defined as ‘the interpersonal attributes exhibited, and teaching approaches used by osteopathy clinical educators in a student-led clinical learning environment’. The present study continues developing the validity argument of a measure of quality of clinical teaching – the Osteopathy Clinical Teaching Questionnaire (OCTQ) [39]. Specifically, the study evaluates clinical educator self-efficacy, in context, and its relationship to self- and student perception of quality of clinical teaching using the OCTQ. The current study is also part of a larger program of research to develop a validity argument for the tools that might be used in a whole-of-system approach to evaluation of clinical teaching and quality assurance of clinical education in the student-led clinic context. The work presented here explores the intersection of student and self-evaluation data about clinical teaching quality, and its relationship to self-efficacy as one factor that may influence this data.

## Methods

This study was approved by the Victoria University (VU) Human Research Ethics Committee.

### Participants

Students enrolled in year 4 (*n* = 80) and 5 (*n* = 55) of the VU were introduced to the study in a practical skills class (outside of the clinic environment) and provided with copies of the OCTQ. Those students interested in participating were encouraged to, prior to their next clinic session [4], complete the OCTQ for each clinical educator with whom they had worked during the July 2017 to November 2017 teaching period and return it to a secure box in the teaching clinic. Student responses were anonymous, and participation in the study was not a requirement of any academic subject in their programs. The student was not required to identify themselves however they were required to write the name of the clinical educator being rated at the top of the form. Consent to participate was implied by return of the questionnaire.

Clinical educators (*n* = 42) employed in the osteopathy program at VU during the same period were invited to complete the questionnaires (OCTQ and SECT), in their own time, in November 2017. Those who chose to participate in the study were asked to identify themselves by name in order to match their self-evaluation data with that obtained from the students. Each clinical educator returned the completed questionnaires to a locked box with consent implied by return of the questionnaire. Only the author had access to the identifiable data and had no role in employment or promotion decisions for clinical educators in the program. The participating clinical educator cohort data summary was made available to the academic clinic coordinator – no data identifying an individual clinical educator was included in this summary.

### Measures

#### Students

Students were asked to complete the Osteopathy Clinical Teaching Questionnaire (OCTQ) for each clinical educator they had worked with during the July to November 2017 period. The Osteopathy Clinical Teaching Questionnaire (OCTQ) was developed to evaluate student perceptions of the quality of clinical teaching in their respective programs’ in student-led, on-campus clinics [40], or university clinics [41]. Previous work provided evidence for the validity argument for the interpretation of scores derived from the OCTQ, including reliability (internal structure, test-retest, inter-rater), content validity, and structural validity [39, 42, 43]. The questionnaire uses a Likert-type scale (strongly disagree (1) to strongly agree (5), with a neutral category) to allow students to respond to each statement. Questionnaires were completed in early November 2017.

#### Clinical educators

The clinical educators were asked to complete:

- a self-evaluation version of the OCTQ containing the same 12 items and 1 global rating item. The anchor for each item was “As a Clinical Educator I …” and items were rephrased to reflect self-rating [1].
- the Self Efficacy of Clinical Teachers (SECT) tool. The SECT tool was developed by McArthur [44] to evaluate self-efficacy of Australian general practice clinical educators, however, the items appear to be suitable for measurement of self-efficacy in the student-led clinical learning environment. The tool contains 22 items across three domains of clinical teaching practice: Customising Teaching to Learning Needs; Teaching Prowess; and, Impact on Learner’s Development, with a total score created for each domain. The Cronbach’s alpha for the 22-item SECT is reported at 0.95 [44].
- a brief demographic questionnaire asking their age, years of practice as an osteopath, years as a clinical educator and whether they had completed a formal university qualification in teaching and learning and/or clinical education.

### Data analysis

Data were entered into SPSS (IBM Corp, USA) for analysis. Total scores were generated for the student evaluations (the OCTQ) and a total score for the clinical educator’s self-evaluation (OCTQ) and also for each of the SECT subscales. Descriptive statistics were generated for the OCTQ completed by the students and the clinical educators, and for the SECT completed by the clinical educators. A difference score was calculated between the student OCTQ scores and the educators self-evaluation OCTQ for both total score and mean. This resulted in a range of scores whereby higher difference scores represented the clinical educator having a higher self-evaluation score than that reported by the students on the OCTQ. Difference scores were then categorized as *higher*, *neutral* and *lower*. Non-parametric tests were used to investigate differences between the demographic variables and the educators’ self-evaluation OCTQ total score, global rating (5-point Likert-type scale) and their SECT subscale scores. Non-parametric effect sizes (r) [6] were calculated where relevant.

#### Relationship between student and clinical educator ratings

Correlations between the student’s OCTQ and educators self-OCTQ ratings were explored with Spearman’s rho (ρ) using the median values for each item, and the global rating item. The relationship between the the educator’s self-evaluation OCTQ, the SECT and the global rating were explored using Spearman’s rho (ρ) and interpreted according to Hinkle, Wiersma and Jurs [45]: 0–0.30 (negligible); 0.30–0.50 (low); 0.50–0.70 (moderate); 0.70–0.90 (high); 0.90–1.00 (very high).

#### Reliability estimates

Reliability estimates for the student OCTQ evaluations were calculated in R [46] using the the MBESS package [47]. Cronbach’s alpha (α) and McDonald’s omega total (ωt), and their respective confidence intervals were calculated consistent with Vaughan [42].

## Results

Three hundred and eight student ratings of a cohort of 42 out of 43 clinical educators who had worked in the student-led, on-campus clinic during the July 2017 to November 2017 teaching period were received. Of the 43 clinical educators, 37 chose to participate in the study including one educator who did not receive student evaluations.

### Demographics

**Fig. 1:**
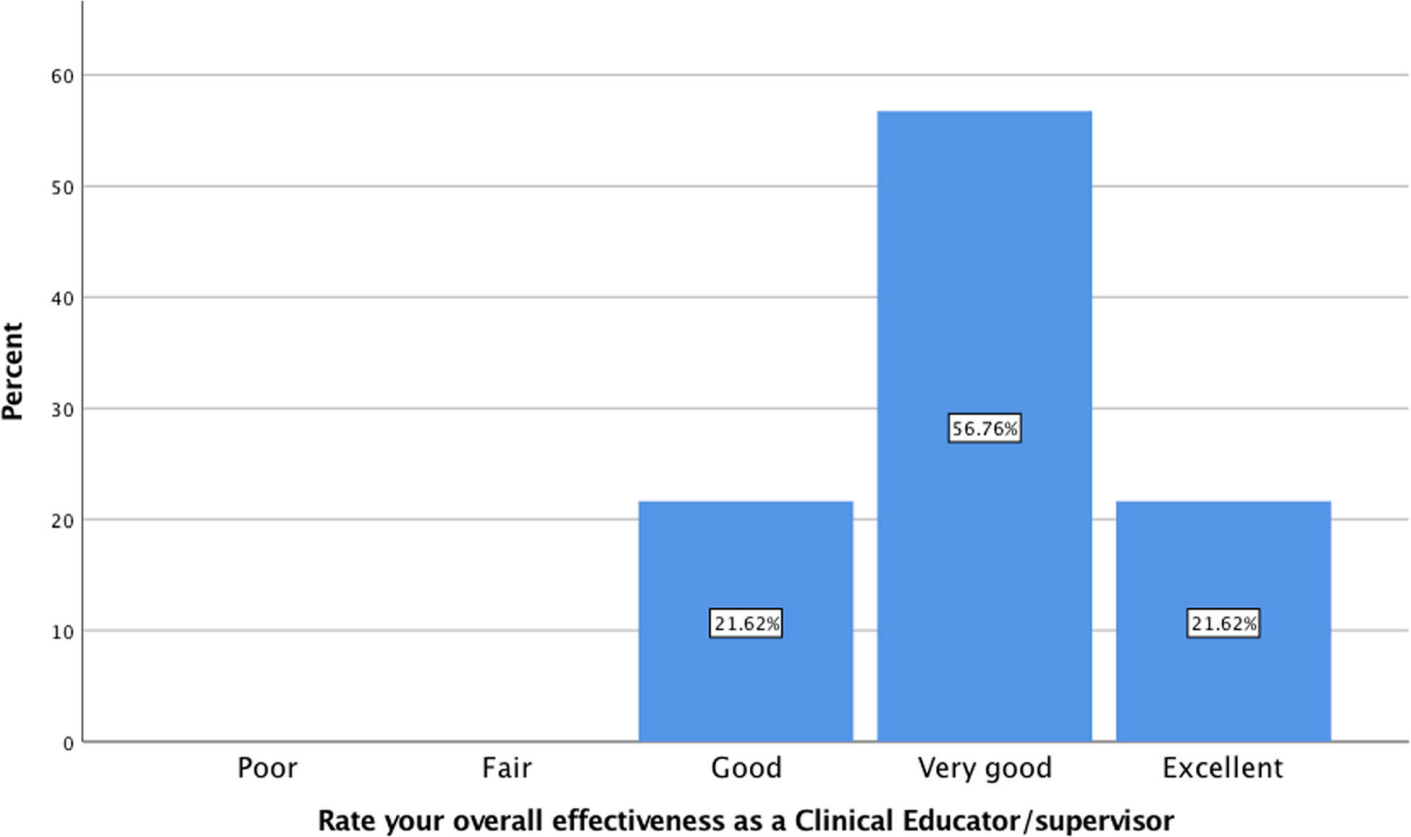
Self-reported overall clinical educator effectiveness

Table 1 presents demographic data for the clinical educators who chose to participate. Table 2 presents descriptive statistics for the student and clinical educator’s self-evaluation versions of the OCTQ for comparison. The mean number of student ratings per educator was 6.75 ± 4.06 with a median of 6 (range 1–14). Clinical educators demonstrated lower means and the same or lower median values for most items when compared to the students. Figure 1 presents clinical educators self-evaluation of overall teaching quality with over 75% rating their effectiveness as *very good* or *excellent*. Table 3 presents the descriptive statistics for the SECT. No significant difference (*p* > 0.05) was identified for any gender, age, years in clinical practice, years clinical teaching, and qualifications for the OCTQ self-evaluation total score, global rating or SECT subscale scores suggesting these variables were not associated with teaching or self-efficacy scores. The reliability estimations for the OCTQ were: ωt = 0.93 [95%CI 0.92–0.95]; and, α = 0.93 [0.91–0.95]. For the SECT, the reliability estimations were: Customising Teaching to Learning Needs subscale (ωt = 0.88 [95%CI 0.82–0.95], α = 0.87 [95%CI 0.79–0.93]); Teaching Prowess subscale (ωt = 0.86 [95%CI 0.80–0.91], α = 0.85 [95%CI 0.79–0.90]; and, Impact on Learner’s Development subscale (ωt = 0.83 [95%CI 0.73–0.92], α = 0.82 [95%CI 0.71–0.89]).

**Table 1:** Demographic characteristics of the clinical educators

**Age**	
25–34 years	18 (48.6%)
35–40 years	11 (29.7%)
41–50 years	6 (16.2%)
51–60 years	1 (2.7%)
Greater than 60 years	1 (2.7%)
**Gender**	
Male	14 (37.8%)
Female	23 (62.2%)
**Years practicing as an osteopath**	
0–4 years	0
5–9 years	20 (54.1%)
10–14 years	9 (24.3%)
15–19 years	6 (16.2%)
20 or more years	2 (5.4%)
**Years as a clinical educator**	
0–4 years	26 (70.4%)
5–9 years	8 (21.6%)
10–14 years	1 (2.7%)
15–19 years	1 (2.7%)
20 or more years	1 (2.7%)
**University teaching and learning qualification**	
Yes	11 (29.7%)
Currently completing	8 (20.0%)
No	18 (48.6%)
**University clinical education qualification**	
Currently completing	1 (2.7%)
No	36 (97.3%)

**Table 2:** Descriptive statistics for the Osteopathy Clinical Teaching Questionnaire completed by the students and clinical educators

Item	Student			Clinical Educator		
	Mean	St Dev	Median	Mean	St Dev	Median
1. Maintain a positive attitude towards students	4.66	0.64	5	4.43	0.60	4
2. Demonstrate humanistic attitudes in relating to patients (integrity, compassion and respect)	4.70	0.57	5	4.73	0.45	5
3 Show genuine concern for my students professional well-being	4.60	0.71	5	4.57	0.50	5
4. Have good communication skills	4.59	0.66	5	4.43	0.50	4
5. Am open to student questions and alternative approaches to patient management	4.55	0.74	5	4.43	0.60	4
6. Adjust teaching to my student’s needs (experience, competence, interest)	4.38	0.82	5	4.14	0.58	4
7. Promote reflection on clinical practice	4.38	0.82	5	4.19	0.66	4
8. Emphasise a problem-solving approach rather than solutions	4.46	0.80	5	4.19	0.87	5
9. Ask questions to enhance my students learning	4.39	0.81	5	4.30	0.66	4
10. Stimulate student’s to learn independently	4.33	0.81	5	4.14	0.79	4
11. Offer my student’s suggestions for improvement when required	4.48	0.82	5	4.32	0.58	4
12. Demonstrated osteopathic, clinical examination and rehabilitation knowledge and skill(s)^a^	3.66	0.67	4	4.27	0.51	4
Total score	53.14	6.99	55	51.34	4.11	50
Global rating	4.39	0.85	5	3.80	0.55	4

^a^ rescored for students only according to Vaughan [42]

**Table 3:** Descriptive statistics for the Self-efficacy in Clinical Teachers (SECT) tool

SECT item	Mean	St Dev	Median
1. I can correctly appraise the learning needs of students	5.16	0.83	5
2. I can write individualised learning objectives based on a student’s unique situation	4.97	1.04	5
3. I can provide appropriate instructional content, based on a student’s learning need.	5.27	0.77	5
4. I can select appropriate teaching strategies when encountering different student’s needs.	5.35	0.98	6
5. I can refine teaching content and methods based on a student’s learning needs and confounding factors	5.05	0.97	5
6. I can teach what the student needs to know	5.62	0.72	6
7. I am effective in my clinical training	5.57	0.80	6
8. I am well organised and prepared for the in-practice teaching	5.46	0.87	5
9. I can provide clinical instruction in a clear manner that students can understand	5.68	0.82	6
10. I can correctly demonstrate clinical skills such as management of the patient consultation/interaction	6.08	0.79	6
11. I have the ability to evaluate the effectiveness of a student’s clinical and consulting efforts through direct observation.	5.43	0.76	5
12. I can teach registrars to determine their professional boundaries	5.54	0.80	6
13. I can handle most difficult student questions or situations	5.59	0.83	6
14. I give clear explanations to questions around clinical scenarios	5.70	0.89	6
15. I can tailor my feedback to be constructive and developmental	5.62	0.92	6
16. I am concerned for my students wellbeing	6.27	0.80	6
17. I have the ability to change the attitude/values of a student	4.95	0.91	5
18. I can design teaching plans for students	4.84	0.99	5
19. I can prepare learning objectives across a student’s area of development	4.86	0.95	5
20. I can give instruction on strategies and resources in a student’s area of development	5.19	0.99	5
21. I can stimulate the student to learn areas of curriculum that don’t interest them	4.92	1.04	5
22. I can provide appropriate support for helping students learn and sustain work/life/family balance and personal wellbeing	5.73	1.02	6
SECT Domain 1 - Customising Teaching to Learning Needs	42.46	5.15	43
SECT Domain 2 - Teaching Prowess	45.92	4.67	47
SECT Domain 3 - Impact on Learner’s Development	30.49	4.28	30

### Difference score

Twenty-four educators (66.7%) had a *lower* difference score (i.e. clinical educator self-OCTQ was less than student OCTQ score) and eleven (30.6%) had a *higher* difference score with one educator (2.8%) demonstrating equal scores. The median difference score was − 1.95 (range − 12 to 16) and no significant difference was identified for the number of student ratings per educator and the difference score category. Age, gender, years practicing as an osteopath, years as a clinical educator and university education qualification were not significantly different for the difference score category. A significant difference was identified between those who had/had not completed a university clinical teaching qualification and difference score (χ^2^ = 35.0, *p* < 0.01). This result suggests that completion of a university teaching qualification may be associated with higher student evaluations compared to those who haven’t completed the qualification. Of note is that there is only one educator currently completing a university clinical education qualification, and this individual educator also demonstrated no difference score, that is, their self and student OCTQ evaluations were equal supporting the aforementioned observation.

As only one educator had no difference score they were excluded from the following analyses. Those educators with a *higher* difference score demonstrated significantly higher total scores for all three SECT domains (Customising Teaching to Learning Needs (Domain 1) – *p* = 0.01, z = − 2.49, *r =* 0.42; Teaching Prowess (Domain 2) – *p* < 0.01, z = − 2.83, *r =* 0.48; Impact on Learner’s Development (Domain 3) – *p <* 0.01, z = − 2.68, *r =* 0.46). These educators were also more likely to rate their global effectiveness as an educator significantly higher with a large effect size (*p <* 0.01, z = − 3.43, *r =* 0.58).

### Relationship between student evaluations and clinical educator self-evaluations

**Fig. 2:**
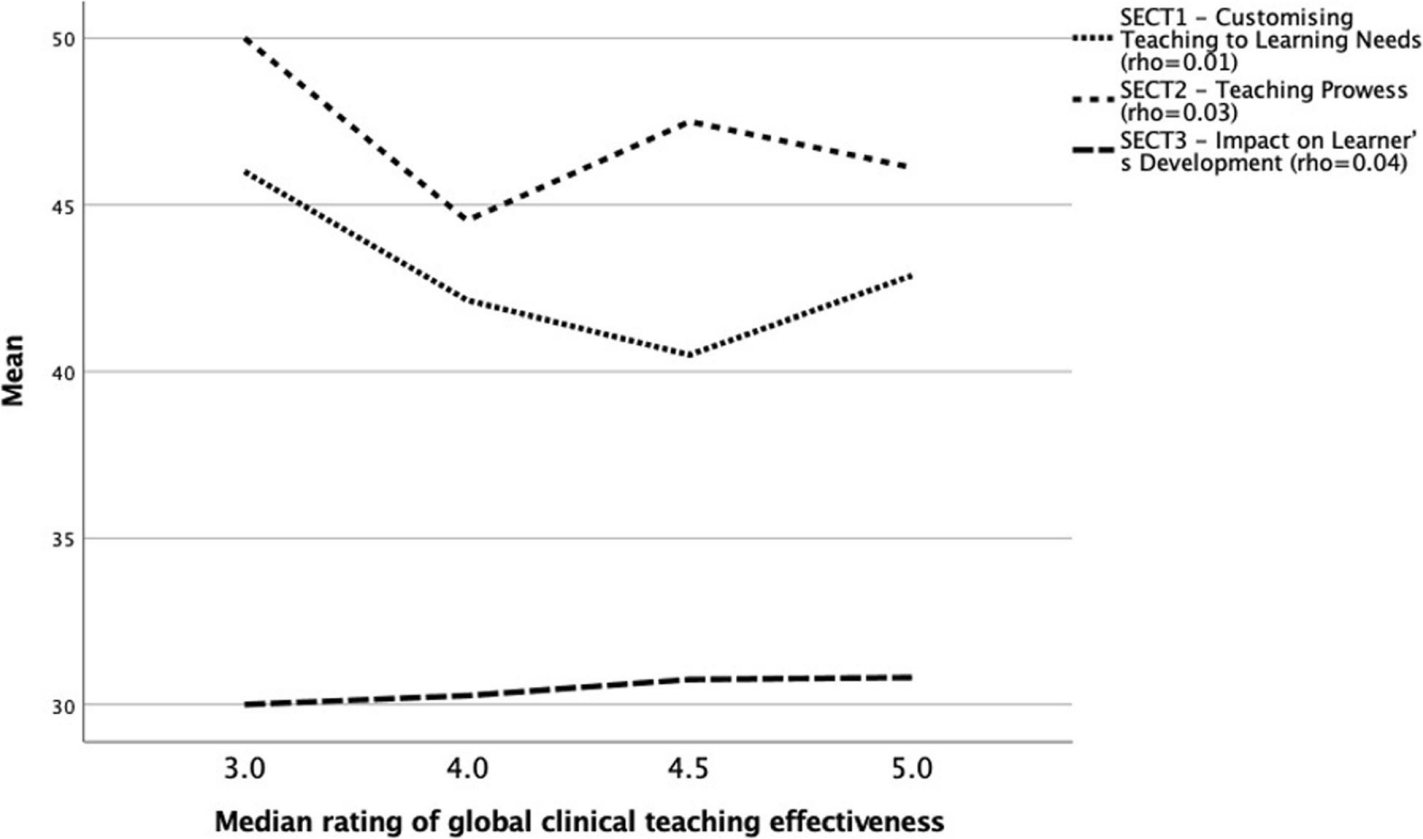
Student median global rating of teacher effectivess and its association with clinical educator self-efficacy

Table 4 presents the relationship between the student and self-evaluation responses to the OCTQ items. Most of the relationships were *negligible*. The relationship between the mean values for item 8 “Emphasises a problem-solving approach rather than solutions” was *low*. The shared common variance for each item ranged from 0.01 to 11.6% suggesting there is little concordance between student evaluations and clinical educator evaluations. Figure 2 shows the associations between student global rating of clinical teaching effectiveness and SECT domains, all of which were trivial and again supporting the limited concordance assertion. Associations between clinical educator completed measures are described in Table 5 with most being moderately correlated except for SECT Domain 3 - Impact on Learner’s Development and the OCTQ self-evaluation total score where a small correlation was observed.

**Table 4:** Association between Osteopathy Clinical Teaching Questionnaire student and self-evaluation

Self-evaluation	Student	Common Variance
1. Maintain a positive attitude towards students	−0.04	0.1%
2. Demonstrate humanistic attitudes in relating to patients (integrity, compassion and respect)	−0.01	0.01%
3 Show genuine concern for my students professional well-being	0.12	1.4%
4. Have good communication skills	−0.12	1.4%
5. Am open to student questions and alternative approaches to patient management	0.22	4.8%
6. Adjust teaching to my student’s needs (experience, competence, interest)	−0.19	3.6%
7. Promote reflection on clinical practice	−0.20	4%
8. Emphasise a problem-solving approach rather than solutions	0.34*	11.6%
9. Ask questions to enhance my students learning	−0.25	6.2%
10. Stimulate student’s to learn independently	−0.15	2.2%
11. Offer my student’s suggestions for improvement when required	−0.15	2.2%
12. Demonstrated osteopathic, clinical examination and rehabilitation knowledge and skill(s)	0.14	2.0%
Global - Rate your overall effectiveness as a Clinical Educator/supervisor	−0.21	4.4%

**Table 5:** Associations between measures completed by the clinical educators

	OCTQ Total	OCTQ Global	SECT Domain 1	SECT Domain 2	SECT Domain 3
**OCTQ Total**	1				
**OCTQ Global**	0.73*	1			
**SECT Domain 1**	0.52*	0.51*	1		
**SECT Domain 2**	0.62*	0.65*	0.72*	1	
**SECT Domain 3**	0.28	0.46*	0.65*	0.56*	1

*p < 0.001; Customising Teaching to Learning Needs (Domain 1); Teaching Prowess (Domain 2); Impact on Learner’s Development (Domain 3

## Discussion

A whole-of-system approach to evaluation of clinical education quality is one aspect of the wider quality assurance program in any health professions education course. One challenge in implementing this approach is the lack of a gold standard measure of clinical teaching quality. Consequently, clinical educators should be encouraged to engage with multiple sources of feedback to benchmark their current performance level [4, 6], and identify opportunities to improve their performance. For that reason this study explored the intersection between clinical educators’ self-evaluation of clinical teaching quality and self-efficacy, and student perceptions of clinical teaching quality. The current study also extends the work of Stalmeijer, Dolmans, Wolfhagen, Peters, van Coppenolle and Scherpbier [1] on clinical educator self-assessment through the inclusion of self-efficacy, given its relationship to teaching effectiveness measures [28].

### Self- and student evaluation

In the current study, three distinct groups of clinical educators were identified:

- Group 1. Those with student evaluations that were higher than the educator’s self-evaluation;
- Group 2. Those with student evaluations that were lower than the educator’s self-evaluations; and,
- Group 3. Those with student evaluations that were consistent with educator self-evaluation.

In relation to clinical educators’ own views of their performance, the disconnect between self- and external evaluation is not new [1, 3, 6], and this trend appears to be the case in the current clinical educator cohort. The trivial to small relationships at item level between the student- and clinical educator OCTQ self-evaluations suggests the educators may be interpreting the items differently to the students, have differing conceptions of clinical teaching quality, or that the OCTQ is not a suitable self-evaluation measure.

Over- and under-estimation of clinical teaching performance in the current work was similar to that of Boerebach et al. [6]. These authors concluded that there were groups who over- and under-estimated their teaching performance, and that in subsequent evaluation rounds, these differences were ameliorated. As these authors highlighted, whether this was due to enacting feedback received in prior rounds, or matching their self-evaluation to previous resident (student) evaluations, could be debated. The results of Boerebach et al. [6] also support the collection of longitudinal teaching quality data [13], affording the educator an opportunity to enact strategies to improve their teaching in response to previous feedback.

Whilst some of the clinical educator cohort in the present study have received ad-hoc formal or informal feedback on their performance, this did not occur on a consistent basis over the study period. The current study was also the first time clinical educators were asked to formally self-evaluate their clinical teaching. Without feedback, it can be challenging for clinical educators to accurately gauge the effectiveness of their clinical teaching performance [1, 48], and this appears to be borne out in the findings of the current study. How clinical educators use this self- and student-derived performance effectiveness information may be mediated by educators’ clinical teaching self-efficacy.

### Self-efficacy

Those clinical educators who were in group 1 (self-evaluation scores higher than student evaluations) demonstrated significantly higher self-efficacy across all three of the SECT domains. This group of clinical educators self-reported they were able to successfully manage the varying demands of clinical supervision and education in the student-led clinical learning environment. This result may also reflect a level of self-confidence with their own performance as a clinical educator. Less experienced clinical educators, both in a clinical and education sense, have been shown to have less confidence in their performance as a clinical educator [49]. However, experience as an osteopathy clinical educator did not appear to be related to higher self-efficacy in the current work. Self-efficacy is both context- and task-specific and when related to self-confidence, a subset of clinical educators in a clinical teaching context may be more likely to display this confidence through their perceived self-efficacy. However, some students in the current study rated clinical educators with low self-efficacy higher than the educator rated themselves (group 2), potentially suggesting this group of clinical educators may be less confident in their performance in this educational context.

Within Bandura’s framework [24], mastery learning is likely to drive confidence with a task (through success or failure) and therefore higher self-efficacy. In the group of clinical educators that demonstrated high self-efficacy, it may be that they have had more perceived successes, and potentially place increased demands on students beyond the students’ *zone of proximal development*. This may have resulted in lower student evaluation scores - an assertion that requires further investigation. Self-efficacy across the three SECT domains was also moderately positively associated with overall self-evaluated teaching effectiveness, further supporting the self-confidence assertion described previously. Self-efficacy accounted for between 21 and 42% of the overall variance in self-evaluated global teaching effectiveness suggesting self-efficacy plays a role in self-evaluation. The significant variation in self-efficacy in our clinical educator cohort, suggests that self-efficacy could be developed in some educators and tempered in others, potentially through professional/faculty development. Thus the current study provides an argument for the use of clinical teaching self-efficacy evaluation as a basis for developing faculty/professional development programs.

Arah et al. [50] demonstrated that those educators who attend training programs are likely to obtain higher student ratings than those who do not, however, participation in formal education programs did not result in higher ratings in the current study. Participating in a generalist post-graduate university teaching qualification may not be the most suitable program for those wanting to undertake more formal education in the clinical education context. This qualification did not appear to be associated with any of the OCTQ completed by the students and clinical educators, nor the SECT. Conversely, the study identified that the one educator who was completing their formal qualification in clinical education demonstrated a self-evaluation score that is consistent with the students’ ratings, although they were not the highest rated educator in the current population. Whether this clinical educator was more accurate at self-assessing due to their clinical education qualification would require additional exploration. It is also important to note that historically, very little clinical education-specific professional development (beyond workplace orientation) has been made available to the educators in the current work.

### Limitations

It is important to be cognizant of the limitations of the current work and the ability to generalize the results to other osteopathy teaching programs, student-led clinics and clinical education more broadly. Defining the construct of ‘clinical teaching quality’ has reported to be challenging [31], and although a definition is provided in the context of the current work, there is no agreed one defined in the literature [31] and the OCTQ may in fact measure ‘satisfaction’. This may also be an additional limit on the generalizability of the study. There are a number of limitations associated with the cross-sectional design of the study including the data being wholly self-report, recall biases, and potential response biases on the part of the students and educators. The student responses were anonymous and therefore less susceptible to social desirability [51], however clinical educator responses were identifiable, and the high self-efficacy and self-evaluations may be due to this bias.

Additional limitations of the work include the study taking place at a single educational institution, there was no question on the demographic form exploring participation in non-award faculty development in clinical education, and the assumption that the SECT captures the breadth of self-efficacy of clinical teaching in the university-based clinical learning environment. The SECT has only been published within a doctoral thesis and the current study is the first to publish data on its use in the peer-review literature. Additional testing of the SECT will strengthen the argument for its use as a measure of self-efficacy for clinical teachers.

The low number of ratings received by some clinical educators may also bias the results in that the student responses may have been more towards one end of the scale providing a biased picture of performance. That said, a single clinical educator receiving a low number of ratings is reflective of the reality of the learning environment in the current study where the educator-student ratio may be small. Statistically this appeared to have minimal impact but larger numbers would be preferable to provide stronger support for the assertions in this work. The difference in self- and student evaluations could be associated with a differing interpretation of the meaning of the OCTQ items. This provides an interesting avenue for further work to understand how the different stakeholders interpret individual items. The small number of educators participating in the study limited the use of regression models that may have assisted in shedding light on the influence of the demographic variables, particularly the influence of gender, on over- or under-estimation of performance [6].

## Conclusions

A whole-of-system approach to evaluation of clinical education is one aspect of quality assurance in any health profession’s education program. Conceptions of clinical teaching quality are likely to be different between the various stakeholders within the clinical education process: student; educator; patient; faculty; peer; and administrator. This study evaluated how clinical educator’s self-evaluation of teaching intersects with their self-efficacy to ascertain if that matches student evaluation of their teaching. Results identified three possible cohorts: a) low student evaluations with high self-evaluation; b) high student evaluations and low self-evaluation; and c) equal student and self-evaluations. Of note was the relationship of the former two groups to self-efficacy - educators self-evaluating their clincal teaching higher than student ratings reported significantly higher self-efficacy. Professional development may be a valuable means of empowering clinical educators, whose self-efficacy is low or those who did not receive high student evaluations. Those educators who have high self-efficacy and low student evaluations may also be tempered through such activities.

Given there is no gold standard measure of clinical teaching quality, clinical educators should engage with multiple feedback sources to benchmark their current performance level and identify opportunities for improvement. Program administrators are also encouraged to consider longitudinal data collected from multiple data sources when making decisions about teaching quality and performance. To further enhance a whole-of-system approach to evaluation of clinical education, future research will explore patient views of clinical educator effectiveness. Such research may illuminate other factors that could assist clinical educators to improve their practice. The complexity of the potential influences on clinical educator performance and teaching quality, requires multiple data sources to inform formative decisions and professional development.

## Data Availability

The datasets generated and/or analysed during the current study are available in the *figshare* repository, 10.6084/m9.figshare.7963823

## Abbreviations

OCTQ: Osteopathy Clinical Teaching Questionnaire
SECT: Self-efficacy of Clinical Teachers
